# A case report on the implications of unexpressed homosexuality

**DOI:** 10.1192/j.eurpsy.2023.2337

**Published:** 2023-07-19

**Authors:** S. P. Godiawala, M. P. N. Parikh, A. Mazumdar, H. Shah, S. Lakhotia, S. Pandey

**Affiliations:** Psychiatry, BJ Medical College, Ahmedabad, India

## Abstract

**Introduction:**

Sexual orientation is one’s romantic preference of attraction, may it be towards the same gender or the opposite. Since ages, lot of communities have considered orientation other than heterosexuality as a taboo. Possession Trance disorder is a trance state in which there is a marked alteration in the individual’s state of consciousness and customary sense of personal identity is replaced by an external ‘possessing’ identity and in which the individual’s behavior and movements are experienced as being controlled by the possessing agent as per ICD 11. While lot of theories for such disorder are established, core of each theory lies at an unconscious underlying conflict that is not acceptable by individual’s psyche. Here is an interesting case of 30yrs old homosexual female having possession trance disorder.

**Objectives:**

To discuss a rare case of possession trance disorder due to unconscious conflict secondary to unexpressed sexual orientation.

**Methods:**

A 30yrs old married female patient diagnosed with Possession Trance disorder as per ICD-11 was on treatment for the same since 3 yrs without improvement. She used to get possession episodes by a religious leader for few hours and would preach to his followers during such episodes. Later she was admitted in indoor facility to understand and explore her illness so as to provide an effective management. After serial interview with the patient and her relatives it was discovered that she had sexual orientation towards females(homosexual). Later on, during the course it was found that patient was attracted to a female disciple of that religious leader and to spend time with her, she used to get possession episodes. This however was not acceptable socio-culturally and by patient herself. This lead to lot of conflicts and dysfunctional marital life with husband. To begin with, patient was unable to accept this fact and reported intense guilt for the same. After serial psychotherapy sessions and pharmacotherapy, she improved significantly. Family based interventions for comprehensive improvement were carried out and the patient was discharged with significant improvement.

**Results:**

Discussion: Lot of communities still has immense stigma against homosexual orientation, at times upto extent to consider it to be some mental illness or supernatural interference. This leads to severe psychological trauma to the person and gives rise to inner conflicts in accepting the true self. This emphasizes a need to develop awareness amongst the communities.

**Conclusions:**

This was an interesting rare case highlighting the need for community based interventions to normalize issues related to human sexuality. There is a need to bring awareness and involvement of community to improve mental health of individual as well as community.

**Disclosure of Interest:**

None Declared

